# Systematic Identification and Comparison of the Expressed Profiles of Exosomal MiRNAs in Pigs Infected with NADC30-like PRRSV Strain

**DOI:** 10.3390/ani13050876

**Published:** 2023-02-28

**Authors:** Feng Cheng, Hui Wang, Lei Zhou, Ganqiu Lan, Hanchun Yang, Lixian Wang, Ligang Wang, Jing Liang

**Affiliations:** 1 College of Animal Science and Technology, Guangxi University, Nanning 530004, China; 2 Key Laboratory of Farm Animal Genetic Resources and Germplasm Innovation of Ministry of Agriculture of China, Institute of Animal Science, Chinese Academy of Agricultural Sciences, Beijing 100193, China; 3 College of Veterinary Medicine, China Agricultural University, Beijing 100193, China

**Keywords:** PRRSV, serum exosome, miRNAs

## Abstract

**Simple Summary:**

Exosomes play a unique role in virus infection, antigen presentation, and suppression/promotion of body immunity. Porcine reproductive and respiratory syndrome virus (PRRSV) is one of the most damaging pathogens in the pig industry. Here, we used the PRRSV NADC30-like CHsx1401 strain to artificially infect 42-day-old pigs, isolate serum exosomes, and identify 33 significantly differentially expressed (DE) exosomal miRNAs between infection and control groups, and 18 DE miRNAs associated with PRRSV infection and immunity were screened as potential functional molecules involved in the regulation of PRRSV virus infection by exosomes.

**Abstract:**

Exosomes are biological vesicles secreted and released by cells that act as mediators of intercellular communication and play a unique role in virus infection, antigen presentation, and suppression/promotion of body immunity. Porcine reproductive and respiratory syndrome virus (PRRSV) is one of the most damaging pathogens in the pig industry and can cause reproductive disorders in sows, respiratory diseases in pigs, reduced growth performance, and other diseases leading to pig mortality. In this study, we used the PRRSV NADC30-like CHsx1401 strain to artificially infect 42-day-old pigs and isolate serum exosomes. Based on high-throughput sequencing technology, 305 miRNAs were identified in serum exosomes before and after infection, among which 33 miRNAs were significantly differentially expressed between groups (13 relatively upregulated and 20 relatively downregulated). Sequence conservation analysis of the CHsx1401 genome identified 8 conserved regions, of which a total of 16 differentially expressed (DE) miRNAs were predicted to bind to the conserved region closest to the 3′ UTR of the CHsx1401 genome, including 5 DE miRNAs capable of binding to the CHsx1401 3′ UTR (ssc-miR-34c, ssc-miR-375, ssc-miR-378, ssc-miR-486, ssc-miR-6529). Further analysis revealed that the target genes of differentially expressed miRNAs were widely involved in exosomal function-related and innate immunity-related signaling pathways, and 18 DE miRNAs (ssc-miR-4331-3p, ssc-miR-744, ssc-miR-320, ssc-miR-10b, ssc-miR-124a, ssc-miR-128, etc.) associated with PRRSV infection and immunity were screened as potential functional molecules involved in the regulation of PRRSV virus infection by exosomes.

## 1. Introduction

Porcine reproductive and respiratory syndrome virus (PRRSV) is a single-stranded positive-strand RNA virus with an envelope structure belonging to the order Nidovirales, family Arteriviridae, genus Betaarterivirus [1,2]. It is spherical or ellipsoidal with a diameter of 50–65 nm under a freezing electron microscope [3,4]. The PRRSV genome is about 15 kb in length with a 5′ cap and a 3′ polyA-tail and contains at least 10 open reading frames (ORFs) flanked by untranslated regions (UTRs) at both the 5′ and 3′ termini [5,6], and is wrapped by nucleocapsid protein, with lipid double-layer coating to form virus particles.

Exosomes belong to vesicles with monolayer membrane structures and have the same topological structure as cells [7]. The shape is “cup-shaped” or “disc-shaped” under an electron microscope [8,9]. Exosomes can exist in the circulatory system for a long time, and substances in exosomes can be absorbed by adjacent cells or distant receptor cells and then regulate the receptor cells to participate in the exchange of genetic materials between cells [10,11]. They are mainly composed of membrane surface substances and carried contents, including cell surface receptors, membrane proteins, soluble proteins, lipids, RNA (mRNA, miRNA, lncRNA, and viral RNA, etc.), genomic DNA, mitochondrial DNA [12,13,14]. MicroRNAs (miRNAs) are a class of 18–25 nucleotides (nt) evolutionarily conserved endogenous non-coding single-stranded small RNAs, which inhibit the translation process by inducing the degradation of target mRNA or by binding with 3′ UTR of target mRNA, leading to post-transcriptional gene silencing, then regulating the gene expression at the post-transcriptional level [15,16,17]. It is estimated that miRNAs regulate more than 60% of mammalian genes post-transcriptionally [18,19]. MiRNAs play an important role in intercellular communication and can also be used as a potential functional molecule for disease and virus infection, transmission, and defense [20]. A growing number of studies have shown that miRNAs can be present in body fluids, such as saliva, urine, breast milk, and blood, and act through the body’s fluid circulatory system [21,22]. Exosomal miRNAs are considered to be endogenous regulators of gene expression and metabolism and can indicate various pathological conditions [23,24].

Over the past two decades, it has been shown that miRNAs have crucial roles in the regulation of immune cell development, innate immune responses, and acquired immune responses. Some other miRNAs are reported to impair PRRSV infection through the following ways, directly target the PRRSV genome or PRRSV receptor, or play a role by regulating the host’s innate immune response. The miR-26 family can significantly damage virus replication, and miR-26a can inhibit the replication of type 1 and type 2 PRRSV strains in porcine alveolar macrophages (PAMs) by regulating the type I interferon (IFN) pathway, which is more efficient than miR-26b [25,26]. miR-30c and miR-125b are identified to modulate host innate immune response by targeting the type I IFN pathway and NF-κB pathway, respectively [27,28,29]. MiR-23, miR-378, and miR-505 are antiviral host factors targeting PRRSV and have conservative target sites in type 2 PRRSV strains [30]. At the same time, host miR-506 has been identified to inhibit PRRSV replication by directly targeting PRRSV receptor CD151 in MARC-145 cells [31]. miR-181 also can indirectly inhibit PRRSV replication by down-regulating PRRSV receptor CD163 in blood monocytes and PAMs [32]. In addition, miRNAs can promote PRRSV replication by interfering with basic cell physiology. MiR-24-3p and miR-22 directly target 3′UTR of HO-1 during PRRSV infection to escape the inhibition of heme oxygenase-1 (HO-1), a heat shock protein (also known as HSP32) on PRRSV [33,34].

Pigs are known to be more susceptible to PRRSV and less able to defend themselves against the entry of this pathogen into the organism [35]. In the present study, the innate immunity and acquired immunity of pigs infected with this virus were studied at the molecular level using a strain prevalent in the field. A serum exosome isolation kit, transmission electron microscopy (TEM), nanoparticle tracking analysis (NTA), and Western blot (WB) were used to isolate and identify serum exosomes before and after infection with PRRSV, followed by small RNA sequencing analysis, identification, and analysis of differential expression results using bioinformatics methods to obtain a number of PRRSV-associated serum exosome miRNAs, followed by identification of data results using quantitative real-time PCR (qRT-PCR).

## 2. Materials and Methods

### 2.1. Animal Experiments

Six PRRSV antigen and antibody double-negative healthy 42-day-old large white pigs were placed in the pig clean feeding system for isolation, healthcare, and environmental adaptation. All pigs were free to eat and drink without restrictions. When they were familiar with the conditions in the isolator, the pigs were nasally inoculated with 2 mL 10^5^ TCID_50_/mL PRRSV NADC30-like CHsx1401, which was mentioned by predecessors [36,37]. The blood of the pigs before (control group, *n* = 6) and 7 days after (treatment group, *n* = 6) virus inoculation was collected from the anterior vena cava for serum isolation. The cellular debris in the serum was removed by centrifugation at 3000 g for 15 min. All animal experiments in our study were approved by the Animal Ethics Committee of the Institute of Animal Science, Chinese Academy of Agricultural Sciences (CAAS) (Beijing, China), IAS2022-130.

### 2.2. Isolation and Purification of Serum Exosomes

Exosome isolation and purification were carried out using the exoEasy Maxi kit (QIAGEN, Hilden, Germany, cat. no. 76064) according to the manufacturer’s protocol.

### 2.3. Transmission Electron Microscopy (TEM)

Extracted exosome suspensions were spotted onto the formvar carbo-coated copper mesh, and the exosomes were rinsed with PBS and subjected to standard uranyl acetate staining for 3 min at room temperature. After drying for several minutes at room temperature, the grid was visualized and photographed at 100 kV by transmission electron microscope (HT-7700, Hitachi-High Tech, Tokyo, Japan).

### 2.4. Nanoparticle Tracking Analysis (NTA)

Extracted exosomes were diluted with 1 × PBS by changing the volume from 10 to 30 μL. After the sample was tested, the concentration and size of serum exosomes were analyzed by an N30E flow nano-analyzer following the manufacturer’s instructions (NanoFCM, Xiamen, China).

### 2.5. Western Blot

The extracted exosome samples were added to RIPA lysate mixed with protease inhibitor (Invitrogen, Waltham, MA, USA) and phenylmethylsulfonyl fluoride (PMSF) to extract the exosome protein, which was lysed on ice for 30 min. Then, according to the instructions of the Bradford kit, we quantified the concentration of serum exosome protein. Exosome proteins underwent thermal denaturation. The same amount of protein was separated on 12% SDS-PAGE gel and then transferred to a polyvinylidene fluoride (PVDF) membrane (Millipore, Burlington, MA, USA). It was soaked in TBST containing 5% skimmed milk powder and sealed for 1 h at room temperature. We soaked the membrane in the diluted primary antibody (anti-CD9 antibody, Abcam, Boston, MA, USA, #ab92726; anti-CD81 antibody, Abcam, Boston, MA, USA, #ab109201) overnight at 4 °C, and recovered the primary antibody. We soaked the membrane in the diluted secondary antibody, incubated it at room temperature for 1 h, and recovered the secondary antibody. We laid the washed film of PBST on the fresh-keeping film, added equal volume mixed ECL a/b chromogenic solution, and placed it in the chemiluminescence imager.

### 2.6. Exosomal Small RNA Sequencing and Data Analyses

Total RNA from the exosomes was extracted with Trizol according to the manufacturer’s instructions. We then detected the RNA concentration and optical density (OD) value and detected the degradation and purity of RNA with 1% agarose gel electrophoresis. Meanwhile, Agilent Bioanalyzer 2100 was used to detect the integrity of RNA. We used the total RNA of exosomes after quality inspection. According to the manufacturer’s instructions, we used NEB NEXT multiplex small RNA library prep set for Illumina^®^ (Illumina, San Diego, CA, USA). The kit prepared a small RNA cDNA library and sequenced it to produce 50 nt single-end reads by the Illumina Novaseq 6000 platform. All the procedures for small RNA library preparation were accomplished by Novogene (Beijing, China).

The data after quality control were aligned to the porcine reference genome (Sus scrofa 11.1) using bowtie. Known miRNAs were identified by the miRbase (v22.0) database [38] (https://www.mirbase.org, accessed on 14 January 2022), miRdeep2 (v0.0.5) [39], and miRevo (v1.1) [40] and were used to predict new miRNAs. At the same time, the differential expression analysis for miRNAs was performed by DESeq (v1.24.0) [41], requiring |fold change| > 1.6 and *p* < 0.05. Alignment was performed using MEGA (V11) [42] followed by single base scoring using PHAST (v1.6.9) [43] and evaluation of the most conserved regions of 10 virus genes, including WUH3 (GenBank accession no. HM853973), VR2332 (GenBank accession no. U87392), JXA1 (GenBank accession no. EF112445), CH-1a (GenBank accession no. AY032626), NADC30 (GenBank accession no. HN654459), HUN4 (GenBank accession no. EF635006), HLJZD22-1812 (GenBank accession no. MN648450), SC/DJY (GenBank accession no. MT075480), and Lelystad (GenBank accession no. M96262.2). RNAhybrid (V2.0) [44] was used to predict the binding of the identified miRNA sequence to the 3′ UTR of the CHsx1401 virus genome. MiRanda (v3.3a) and RNAhybrid were used to target gene prediction. The clusterProfiler [45] R package was used for GO (Gene Ontology) functional enrichment analysis of target genes and KEGG (Kyoto Encyclopedia of Genes and Genomes) pathway enrichment analysis.

### 2.7. Validation of miRNA Expression by RT-qPCR

Total RNA was isolated from serum exosomes using Trizol (Invitrogen, Shanghai, China) according to the manufacturer’s protocol. The isolated RNA was verified by RT-qPCR on samples (*n* = 6 per group). cDNA was synthesized according to the instructions of miRNA 1st strand cDNA synthesis (by stem-loop) kit (Vazyme, Nanjing, China), and the fluorescence quantification was performed using ABI 7500 according to the instructions of miRNA universal SYBR qPCR master mix (Vazyme, Nanjing, China). The thermal cycle parameters used were as follows: the first stage: 95 °C for 30 s; Stage 2: 95 °C for 5 s, 60 °C for 34 s, and 40 cycles; Stage 3: 95 °C for 15 s, 60 °C for 1 min, and 95 °C for 15 s. Primer sequences of miRNAs, the U6 gene, were used as a reference [46] and listed in Supplementary Table S1. All qRT-PCR verifications were performed using three biological replicates and with three replicates for each sample. The relative abundance of transcripts was calculated by the 2^−ΔΔCt^ method, and SPSS (v22.0) and GraphPad Prism (v8.0) were used for data analysis and mapping, respectively. *p* < 0.05 means the difference is statistically significant.

## 3. Results

### 3.1. Relative Value of Antigen and Antibody after Virus Inoculation

The results of PRRSV antigen and antibody tests before (day 0) and after the (day 7) challenge are shown in Table 1. The serological detection of the PRRSV antigen and antibody before the challenge was negative, and the antigen was positive after the challenge, indicating that the pigs were successfully infected with CHsx1401.

### 3.2. Isolation and Identification of Serum Exosomes

**Figure 1:**
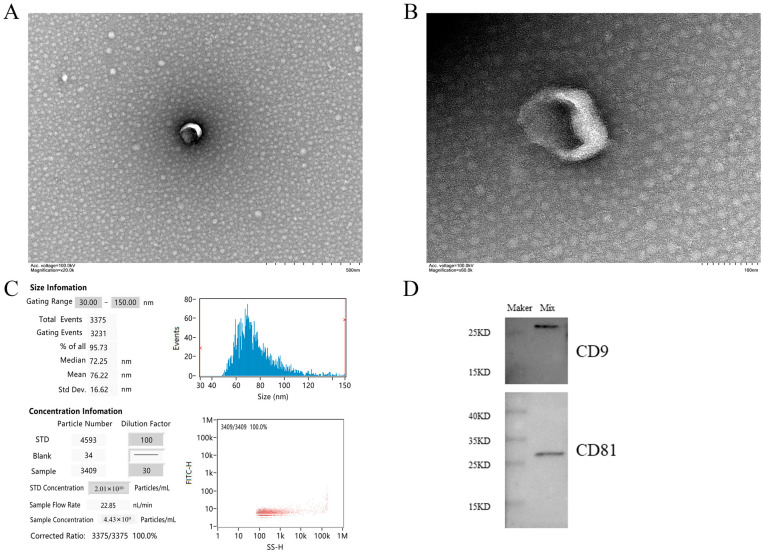
Main characteristics of serum exosomes. (**A**,**B**) show the morphological characteristics of vesicles by TEM. Scale bars are 500 nm and 100 nm, respectively. (**C**) NTA shows the diameter and concentration of most vesicles. (**D**) Western blot showed the presence of exosome markers CD81 and CD9 in serum exosomes. Note: Mix in WB results is the sample mixed suspension isolated by the exoEasy Maxi kit.

The vesicles isolated from serum were discovered by TEM. Most vesicles can clearly see the concave saucer- or disc-shaped exosomes in the middle. The membrane edge of exosomes is clearly visible, and the morphology is relatively complete (Figure 1A,B). The nanoparticle tracking analysis showed that 95.73% of the exosomes had a diameter of 30–150 nm, mainly around 72.25 nm, with an average diameter of 76.22 nm, which was consistent with the size characteristics of exosomes (Figure 1C). This size range was similar to that detected by TEM and further confirmed the identity of these vesicles as exosomes. Western blot analysis showed that the vesicles isolated from the serum samples were positive for CD9 and CD81 proteins (Figure 1D). The above characteristics conform to the exosome identification standards formulated by the international society for extracellular vesicles (ISEV) in MISEV2018 [47].

### 3.3. Small RNA Sequencing of Serum Exosomes

**Figure 2:**
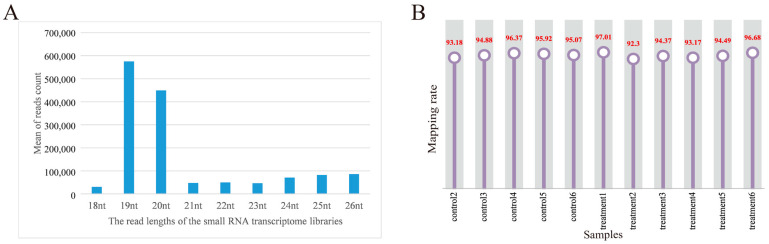
Overview of the small RNA transcriptome data. (**A**) Length distribution of read counts of serum exosome samples (nt = nucleotides); (**B**) rate of 12 samples mapped to the reference genome.

For each sample, the clean data reached 0.5 Gb, and the Q30 base percentage was above 96.20%. The clean reads of each sample were aligned with the pig reference genome. Among the 12 samples, the control group obtained 10,920,887, 10,248,696, 10,109,117, 10,655,494, 9,217,285, and 9,782,523 reads, respectively. The treatment group obtained 11,889,518, 10,593,504, 10,593,504, 12,846,080, 10,105,325, 11,729,451, and 9,789,542 reads, respectively. On average, 77.96% of the total clean reads comprised 19–22 nucleotides (nt) in length (Figure 2A). The reads after quality control accounted for more than 92.59% of the total reads. The processed clean reads were aligned to the porcine reference genome, and the mapped rate of 12 libraries on the genome was more than 92.30%, and the mapped rate was 94.98% (Figure 2B). It indicated that the constructed serum exosomal miRNA library was of high quality and suitable for further analysis. Details are listed in Supplementary Table S2.

### 3.4. Differentially Expression Analysis of miRNAs

**Figure 3:**
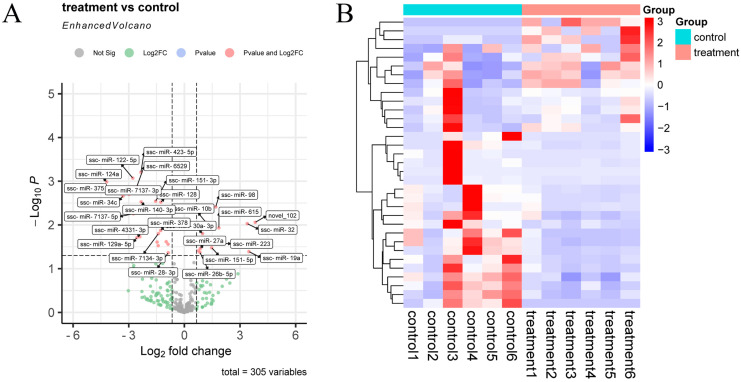
Differential expression of miRNAs in exosomes. (**A**) Volcano plot of miRNAs between control and treatment groups; (**B**) hierarchical clustering heatmap of DE miRNAs between control and treatment groups.

After quantitative analysis of the identified miRNA expression, miRNAs were screened by the thresholds described previously in Section 2.6. A total of 305 miRNAs were obtained before and after inoculation of the CHsx1401 strain (control, *n* = 6; treatment, *n* = 6). A total of 33 differentially expressed (DE) miRNAs were identified between the two groups, 13 DE miRNAs were upregulated, and 20 DE miRNAs were downregulated in the treatment group (Figure 3 and Supplementary Table S3).

### 3.5. Functional Enrichment Analysis of miRNA Target Genes

**Figure 4:**
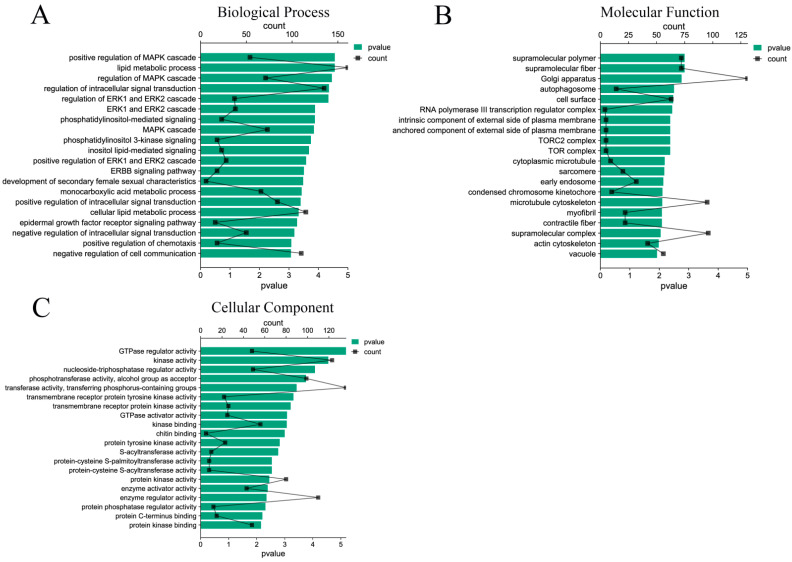
GO function enrichment analysis of DE miRNAs target genes. (**A**) Biological process of DE miRNAs target genes; (**B**) molecular functions of DE miRNAs target genes; (**C**) cellular components of DE miRNAs target genes.

A total of 7283 target genes were predicted by 33 DE miRNAs, and the functions of target genes were mainly concentrated in the positive regulation of MAPK cascade, lipid metabolism process, regulation of intracellular signal transduction, ERK1 and ERK2 cascade, etc. (Figure 4A). In terms of molecular functions, the differentially expressed miRNAs target genes mainly focus on GTP-enzyme regulatory activity, kinase activity, nucleoside triphosphatase regulatory activity, and other functions related to signal transduction and energy metabolism (Figure 4B). In addition, among the cell components, the target genes mainly participate in the biological functions of supramolecular polymers, Golgi, autophagosomes, cell surface, early endosomes, etc. (Figure 4C). The functions of these components are closely related to the formation of exosomes, which also explains the accuracy of the sequencing.

**Figure 5:**
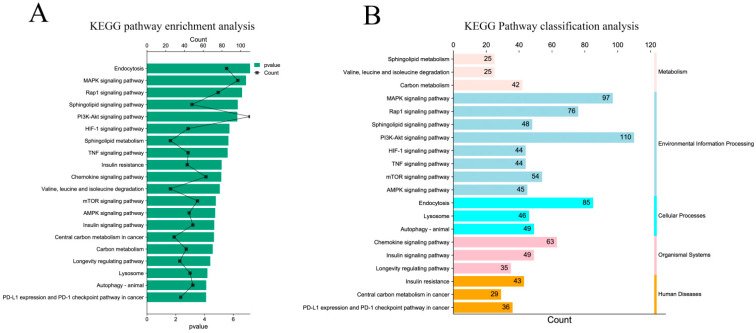
KEGG pathway enrichment analysis of target genes. (**A**) Significantly enriched KEGG pathway with target genes of DE miRNAs; (**B**) classification of significantly enriched KEGG pathways.

KEGG pathway enrichment analysis showed that the target genes were significantly enriched in endocytosis, the MAPK signaling pathway, the Rap1 signaling pathway, the sphingolipid signaling pathway, and the PI3K Akt signaling pathway (*p* < 0.05) (Figure 5A). At the same time, the enriched pathways were classified and analyzed. The results showed that the KEGG pathway of the target gene was mainly enriched in environmental information processing, human diseases, and biological systems (Figure 5B).

### 3.6. Targeting Prediction of Serum Exosomal miRNA and PRRSV CHsx1401 Genome

**Figure 6:**
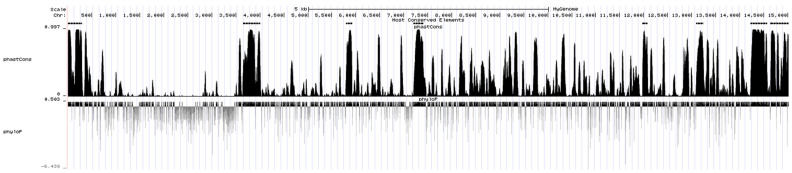
Conserved segments in the genome of CHsx1401 strain predicted by PHAST.

According to the phastCons score of a single base after alignment by PHAST, a total of eight most conserved segments (black bands above the peak map) were obtained among the viral genomes (Figure 6). A total of 31 DE miRNAs were found to bind to the conserved segment by predicting the miRNAs bound to the conserved segment. Among them, in the conserved region (14,644–15,020 nt) closest to the 3′ UTR (14,870–15,020) of CHsx1401 genome, 16 DE miRNAs are predicted to bind to it, including 5 miRNAs (ssc-miR-34c, ssc-miR-375, ssc-miR-378, ssc-miR-486, and ssc-miR-6529) that can bind to the 3′ UTR of CHsx1401. Among these miRNAs, only ssc-miR-223 was upregulated after infection, and other miRNAs were downregulated after infection. See Supplementary Table S4 for details.

### 3.7. Screening DE miRNAs Related to Exosome Function and PRRSV

**Figure 7:**
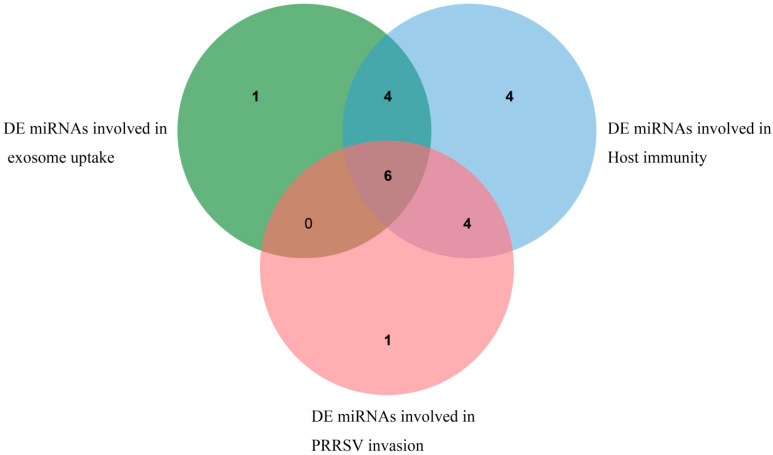
DE miRNAs related to exosome uptake, PRRSV invasion, and immunity.

A variety of differentially expressed miRNAs related to the function of exosomes and PRRSV were found by functional enrichment analysis of target genes. Among them, 11 DE miRNAs such as ssc-miR-4331-3p, ssc-miR-744, and ssc-miR-320 are involved in exosome uptake, and their target genes are mainly concentrated in the Ras gene family, annexin family, and ADP ribosylation gene family. Eighteen DE miRNAs, including ssc-miR-10b, ssc-miR-124a, and ssc-miR-128, participate in immune-related pathways, and their target genes are mainly concentrated in the MAPK gene family, PIK3 gene family, and protein phosphatase gene family. While 11 DE miRNAs are involved in virus invasion, the related target genes are mainly concentrated in the MAPK gene family and protein phosphatase gene family. Furthermore, multiple differentially expressed miRNAs, such as novel_102. Six DE miRNAs, including ssc-miR-320, ssc-miR-423-5p, ssc-miR-4331-3p, ssc-miR-7137-3p, and ssc-miR-744, are co-expressed in exosome function, PRRSV virus invasion, and immune-related pathways, as shown in Figure 7. Details are shown in Supplementary Table S5.

### 3.8. QRT-PCR Assay of DE miRNAs between the Two Groups

**Figure 8:**
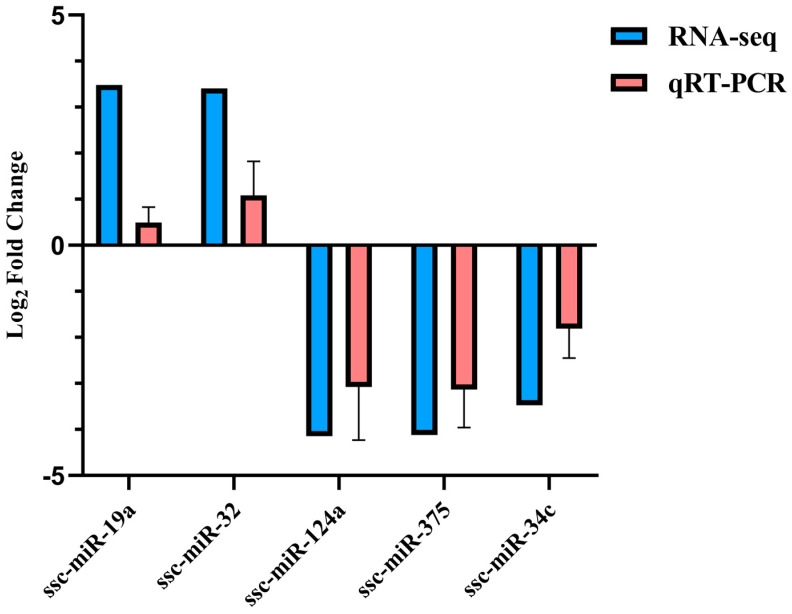
Five DE miRNAs validated by qRT-PCR.

Five DE miRNAs were randomly selected for verification. According to the qRT-PCR results, the expression of ssc-miR-19a and ssc-miR-32 increased in the treatment group, while ssc-miR-124a, ssc-miR-375, and ssc-miR-34c showed higher expression in the control group, consistent with the sequencing data (Figure 8).

## 4. Discussion

PRRSV is still a stubborn pathogen in the global pig industry, causing huge economic losses in the world. At present, vaccination is mainly used to prevent and control PRRSV, among which the modified live (MLV) virus vaccine is the most widely used [48]. Although this vaccine was effective in reducing PRRS outbreaks and incidence, it also greatly increased genetic variation and diversity of the virus and led to viral recombination between wild and live vaccine viruses in the field [49,50]. In recent years, the spread and prevalence of the recombinant virus NADC30-like PRRSV strain have caused multiple outbreaks of porcine reproductive and respiratory syndrome in China. The similarity between CHsx1401 and NADC30 used in this study remained at 92.2–99.1%. Since then, it has become an epidemic strain in China. Exosomes, as mediators of cell communication, are widely found in various body fluids and have unique advantages in disease diagnosis and treatment [51,52]. According to previous reports, exosomes play an important communication role in antigen presentation [53], immune response [53,54], virus replication [54], cancer [55], neurodegenerative diseases [56], angiogenesis [57], tumor cell migration [58] and invasion [59], and have high research value.

In this study, high-throughput sequencing technology was used to construct the miRNA expression profile of serum exosomes, and 33 DE miRNAs were identified. As we all know, the host-encoded miRNA can bind with the viral genome and then regulate the replication, synthesis, and release of the virus to limit infection and affect the pathological process [15]. Studies of miRNAs targeting the viral genome have also been repeatedly reported in animals. gga-miR-454 and gga-miR-130b in chicken infectious bursal disease can target the viral genome to inhibit viral replication, while gga-miR-21 directly targets the viral protein VP1 to inhibit viral protein translation [60,61]. In PRRSV studies, ssc-miR-181 specifically binds to a highly conserved region downstream of the viral genome ORF4 and strongly inhibits PRRSV replication [62]. In this study, the expression difference of ssc-miR-181 between the two groups did not reach a significant level. In our study, the genomes of nine different PRRSV viruses were compared with those of the CHsx1401 strain, and the eight most conserved segments were identified. It was predicted that 31 DE miRNAs could bind to the 8 most conserved segments of CHsx1401, and 16 DE miRNAs could bind to the conserved sequences close to the 3′ UTR of CHsx1401. Among them, 5 DE miRNAs (ssc-miR-34c, ssc-miR-375, ssc-miR-378, ssc-miR-486, and ssc-miR-6529) can simultaneously bind to the CHsx1401 3′ UTR. In addition, the upregulated expression of ssc-miR-223 was predicted to bind to the 3′UTR target of the PRRSV genome. The results showed that the conserved sequences of the virus genome might play a key role in its pathogenicity, and the miRNAs that can bind to the conserved sequences between the genomes of different PRRSV strains may have important significance in controlling the pathogenicity of the virus. Some differentially expressed miRNAs have been proven to be related to PRRSV by previous studies and even directly involved in the regulation of PRRSV, including ssc-miR-10b [63], ssc-miR-378 [30], ssc-miR-124a [64], let-7f-5p [65], ssc-miR-744 [66], and ssc-miR-19a [67].

PRRSV can evade host defense by interfering with innate immune response. This process is regulated by many signaling pathways, including the MAPK signaling pathway, PI3K Akt signaling pathway, autophagy, chemokine, and TNF signaling pathway. At present, the MAPK signaling pathway includes three main pathways: ERK1/2, JNK, and p38 pathway. Activation of the MAPK cascade can promote host cell apoptosis, assist the virus in escaping the host immune defense response and promote PRRSV replication [68]. Moreover, the activation of c-Jun N-terminal kinases (JNKs) and p38 can also promote the release of the inflammatory factor IL-10 [68,69,70] and enhance the inflammatory effect. In addition to inducing apoptosis, PRRSV can also induce autophagy, which can promote PRRSV replication. The activation of PI3K/Akt is necessary for virus entry and promotion of virus replication, and PRRSV-activated Akt inhibits host cell apoptosis by negatively regulating the JNK pathway [71]. TNFα It can play an important role in the induction and regulation of inflammatory response together with other inflammatory factors, but TNF α Expression is affected by the negative regulation of PRRSV replication [72]. In the present study, miRNAs (ssc-miR-10b, ssc-miR-122-5p, ssc-miR-124a, ssc-miR-128, ssc-miR-129a-5p, etc.) enriched in these pathways are involved in PRRSV-induced apoptosis, autophagy, and inflammation and are closely associated with viral immune response, immune evasion, and replication.

The cell plasma membrane is rich in a variety of lipid rafts, and sphingolipid- and cholesterol-rich in sphingolipids (sphingomyelin and glycosphingolipids) are key molecules of lipid rafts. The recognition of lipids by some proteins of the virus may be a necessary condition for the entry of the virus [73]. Envelope viruses insert viral envelope glycoproteins into lipid rafts at the stage of virus entry, interact with receptors located in lipid rafts, or change from their natural state to activated form to initiate or promote viral internalization/fusion, such as HSV, SARS coronavirus, and piglet epidemic diarrhea virus [73,74]. Previous studies found that the removal of cholesterol from the surface of MARC-145 cells significantly reduced PRRSV infection, demonstrating that inhibition of PRRSV infection was specifically mediated by the removal of cellular cholesterol. Depletion of cell membrane cholesterol significantly inhibited virus entry, particularly virus attachment, and release [75]. Obviously, sphingolipid metabolism can regulate membrane structure and adhesion, which is of great significance in PRRSV virus invasion.

Endocytosis was the most significant enrichment in this study. Endocytosis is an important mechanism of exosome uptake by target cells. Previous studies have shown that exosome uptake is an energy-demanding and cytoskeleton-dependent process, which highlights the potential role of endocytosis in this process [76]. It has been proved that there are several pathways that can mediate this process, including phagocytosis, macropinocytosis, clathrin, etc. [77,78], which led to different classifications and roles of endocytosed substances. The enrichment of differentially expressed exosomal miRNAs in this pathway indicates that exosomes play an important role in PRRSV infection, and the regulation of content transport and uptake in exosomes may lead to pathophysiological changes in target cells and organs.

## 5. Conclusions

Through the identification and bioinformatics analysis of serum exosomal miRNAs from PRRSV-infected pigs, a variety of PRRSV-related pathways and differentially expressed miRNAs were obtained in this study, such as ssc-miR-4331-3p, ssc-miR-744, ssc-miR-320, ssc-miR-10b, ssc-miR-124a, ssc-miR-128, etc., which play potential functional roles in PRRSV-induced immune response, invasion, and exosome uptake. In addition, because a single miRNA can target multiple genes and a single gene is also regulated by multiple miRNAs, there are a number of miRNAs that perform multiple functions in the above pathways. Some miRNAs have been verified to regulate PRRSV infection by acting on key receptors or directly targeting the virus genome, such as ssc-miR-10b, ssc-miR-378, miR-124a, let-7f-5p, ssc-miR-744, ssc-miR-19a, etc. Meanwhile, the present study also predicted a variety of miRNAs that can bind to the most conserved fragment of the 3′ UTR of the CHX1401 virus genome, including ssc-miR-34c, ssc-miR-375, ssc-miR-378, ssc-miR-486, and ssc-miR-6529, which may be important for regulating viral pathogenicity.

## Figures and Tables

**Table 1:** Antigen and antibody of (day 0) and (day 7) with challenge virus.

Time	Control/before Inoculation (Day 0)	Treatment/after Inoculation (Day 7)
**Antigen (Ct)**	/	26.05
	/	25.31
	/	20.69
	/	24.70
	/	25.01
	/	22.55
**Antibody (S/P)**	0.03	/
	0.12	/
	0.15	/
	0.06	/
	0.25	/
	0.00	/

Note: antigen “/” means negative, antibody < 0.4 means negative, Ct means cycle threshold, S/P value = (sample value − negative control mean value)/(positive control mean value − negative control mean value).

## Data Availability

The data presented in this study are openly available in NCBI Sequence Read Archive under accession number PRJNA938232.

## Supplementary Materials

The following supporting information can be downloaded at: https://www.mdpi.com/article/10.3390/ani13050876/s1, Table S1: Primer sequence of miRNA for qRT-PCR; Table S2: Length distribution of read counts; Table S3: DE miRNAs in between control and treatment group; Table S4: Predicted binding of differentially expressed miRNA to CHsx1401 genome; Table S5: DE miRNAs related to exosome function and PRRSV.
